# Pyroptosis in platelets: Thrombocytopenia and inflammation

**DOI:** 10.1002/jcla.24852

**Published:** 2023-02-28

**Authors:** Yang Su, Tiannan Zhang, Rui Qiao

**Affiliations:** ^1^ Department of Laboratory Medicine Peking University Third Hospital Beijing China

**Keywords:** caspase‐1, GSDMD, inflammasome, NLRP3, platelets, pyroptosis

## Abstract

**Objective:**

The purpose of this manuscript was to conclude the role of platelets in immune inflammation and discuss the complex mechanisms of pyroptosis in platelets as well as their related diseases.

**Methods:**

This article reviewed the existing literature to see the development of pyroptosis in platelets.

**Results:**

Platelets have been shown to be capable of activating inflammasomes assembled from NOD‐like receptor family pyrin domain containing 3 (NLRP3), apoptosis‐associated speck‐like protein containing a CARD (ASC) and caspase‐1. Recently, they were also implicated in pyroptosis. Cleaved by caspase‐1, N‐terminal gasdermin D (N‐GSDMD) could form pores in the cell membrane, inducing nonselective intracellular substance release. This programmed cell death induced thrombocytopenia and inflammatory cytokine release such as IL‐1β and IL‐18, promoting platelet aggregation, vaso‐occlusion, endothelial permeability and cascaded inflammatory response.

**Conclusion:**

Pyroptosis in platelets contributes to thrombocytopenia and inflammation.

## Graphical abstract

This review describes the complex mechanisms of pyroptosis in platelets and their relative diseases. Platelet NLRP3 activation is induced via LPS, HMGB1 and S100A8/A9 receptor TLR4, collagen receptor GPVI, and thrombin. Upon NLRP3 activation, NLRP3 forms inflammasome complexes with the ASC and pro‐caspase‐1 that regulate the production of proinflammatory cytokines such as IL‐1β and IL‐18, at the same time, cleaving GSDMD and inducing pyroptosis. Mitochondrial‐derived ROS and its upstream of RIP1/RIP3 regulate NLRP3 inflammasome recruitment. Pyroptotic platelet‐derived oxidized mitochondrial DNA (ox‐mtDNA) potentially promotes neutrophil extracellular trap (NET) formation, which contributes to platelet pyroptosis by releasing S100A8/A9, forming a positive feedback loop that led to the excessive release of inflammatory cytokines.

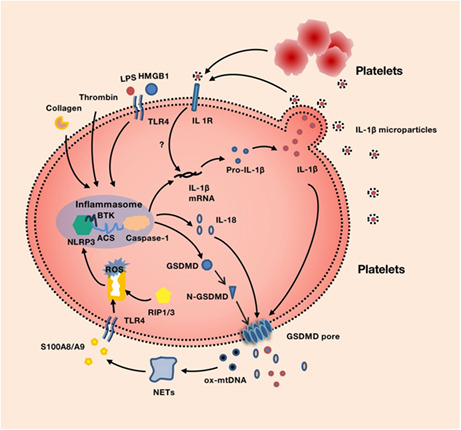

Platelets are anucleated cytoplasmic fragments generated from megakaryocytes in the bone marrow and are cleared in the reticuloendothelial system.^1^ Around one trillion platelets (150–450 × 10^9^/L) circulate in the blood of a healthy individual, outnumbering all other leukocytes in the vasculature by several folds.^2^ In addition to their role in hemostasis and thrombosis, platelets have been increasingly recognized as multipurpose cells involved in processes including tissue repair, immunity, and tumors.^3^ Evidence has proven that platelets are actually a kind of both immune sensing cell and immune effector cell. By interaction with macrophage cells, monocytes, neutrophils, lymphocytes, and the endothelium, platelets are therefore important executors during both inflammatory and immune responses.^4^

To exert these functions, platelets express pattern recognition receptors (PRRs), including Toll‐like receptors (TLRs), Nod‐like receptors (NLRs), and absent in melanoma‐like receptors (ALRs), which play critical roles in sensing and responding to pathogen‐associated molecular patterns (PAMPs) or damage‐associated molecular patterns (DAMPs).^5^ Upon stimulated by PAMPs and DAMPs, platelets are capable of complexing NLRP3 inflammasomes and activating caspase‐1, regulating the production of proinflammatory cytokines such as IL‐1β and IL‐18.^6^ Although platelets do not have nuclei, they have stored RNA molecules and diverse mechanisms for post‐transcriptional processing of RNA using specialized pathways to change their proteome, phenotype, and functions. Therefore, platelets might provide an important crosstalk interface for inflammation and coagulation.^7^

Among this crosstalk function of platelets, pyroptosis, as a type of programmed cell death, may play key roles. Induced by the canonical caspase‐1 inflammasomes responding to PAMPs and DAMPs such as bacterial, viral, toxin, and chemotherapy drugs^8^ or by activation of caspase‐4, ‐5 and ‐11 by cytosolic lipopolysaccharide (LPS),^9^ pyroptosis can result in strong inflammatory responses in infectious and noninfectious diseases. More other pathways have been gradually discovered in recent years. Pyroptotic cells undergo cell osmotic balance destruction, cell swelling, and membrane dissolution, followed by the release of cell contents and cytokines such as IL‐1β and IL‐18, exacerbating the inflammatory response and promoting coagulation.^10^, ^11^ Although pyroptosis usually occurs in professional phagocytes of the myeloid lineage, such as macrophages, dendritic cells, and neutrophils,^12^ it has recently also been found in platelets. In this review, we will summarize the probable mechanisms of pyroptosis in platelets as well as their related diseases.

## 1 PYROPTOSIS

### 1.1 Discovery of pyroptosis

The first relative study on pyroptosis could be tracked to 1986 when Friedlander first observed that treatment of mouse macrophages with anthrax lethal toxin resulted in cell death and rapid release of cell contents.^13^ In 1992, Zychlinsky et al.^14^ confirmed that the cell death caused by the Gram‐negative bacterial pathogen Shigella flexneri in its host macrophages was a form of suicide, yet it was thought to be apoptosis at that time. Two years later, they found that peritoneal macrophages undergoing suicide induced by Shigella flexneri infection could release the inflammatory cytokine interleukin 1 (IL‐1).^15^ Coincidentally, Thornberry et al.^16^ reported that interleukin‐1β‐converting enzyme (ICE) was an inflammatory cysteine protease and novel in the process of cleaving precursor IL‐1β into mature IL‐1β. It is easy to associate the release of IL‐1 in suicidal macrophages induced by Shigella flexneri with ICE. To further confirm this, in 1996, Zychlinsky et al.^14^ found that the invasion plasmid antigen B (IpaB) of Shigella activated macrophage programmed death by binding to ICE, and specific inhibitors of ICE could prevent Shigella‐induced programmed cell death,^17^, ^18^ showing the important role of ICE in this kind of programmed cell death. In case of inconsistent and multiple names, the cysteine protease ICE was termed caspase‐1.^19^ In 1998, Fantuzzi et al.^20^ found that in addition to pro‐IL‐1, pro‐IL‐18 processed by caspase‐1 could lead to the release of bioactive IL‐18. In 1999, Hersh et al. found that suicide macrophages infected by the invasin SipB of Salmonella spp., which functioned as an analog of the Shigella invasin IpaB, were also associated with caspase‐1, as macrophages lacking caspase‐1 were not susceptible to Salmonella‐induced programmed cell death.^21^, ^22^

Increasing findings regarding bacteria‐induced programmed cell death of macrophages have led to the apparent paradoxical conclusion on apoptosis that the induction of programmed cell death in these systems is proinflammatory and caspase‐1‐dependent. This suggested that bacteria‐induced programmed cell death differed from apoptosis seen in the development or maintenance of tissues or organs.^23^ Until 2001, Brad T Cookson and Molly A Brennan named this form of programmed cell death in macrophages infected by Shigella and Salmonella as “pyroptosis”.^24^In 2002, Martinon et al.^25^ reported that inflammasomes comprising caspase‐1, caspase‐5, ASC, and a pyrin domain‐containing protein sharing structural homology with NODs (NALP1) were first considered to activate inflammatory caspases and process pro‐IL‐1β. In 2008, Fink et al.^26^ found that both anthrax lethal toxin and Salmonella induced DNA fragmentation and cell membrane damage, causing intracellular content release and a serious inflammatory reaction in pyroptotic macrophages. In 2011, Kayagaki discovered that caspase‐11 in mouse (known as caspase‐4/5 in human) could also induce caspase‐1 activation, IL‐1β production, and death of macrophages when infected with Escherichia coli, Citrobacter rodentium, or Vibrio cholera.^27^ This process was similar to but not inflammasome‐mediated pyroptosis. To distinguish the two pathways, inflammasome‐mediated pyroptosis was termed the “canonical pyroptosis pathway,” and caspase‐4/5/11‐mediated pyroptosis was termed the “non‐canonical pyroptosis pathway”. And, in 2015, gasdermin D(GSDMD) was found to be the common substrate of caspase‐1 and caspase‐4/5/11, and the N‐terminal domain of cleaving GSDMD (N‐GSDMD) could form pores in the cell membrane, inducing intracellular substances release.^11^, ^28^

### 1.2 Mechanism

#### 1.2.1 Canonical pathway

The canonical pathway of pyroptosis is a kind of caspase‐1‐dependent pathway. The assembly of inflammasomes begins with PRRs, which are capable of recognizing PAMPs and DAMPs.^29^ PRRs (e.g., NLRP1, NLRP3, NLRC4, aim2, etc.) assemble with ASC and pro‐caspase‐1 to form inflammasomes after stimulation by PAMPs and DAMPs.^30^ After inflammasome assembly, pro‐caspase‐1 is activated and is hydrolyzed into two fragments, forming a dimer to become mature cleaved caspase‐1.^31^ Then, caspase‐1 cleaves the connection between the N‐terminal and C‐terminal GSDMD rapidly and forms the 22 kDa C‐terminus (C‐GSDMD) and 31 kDa N‐terminus (N‐GSDMD).^11^ N‐GSDMD perforates the cell membrane to form nonselective GSDMD pores with inner diameters of 10–14 nm, leading to cell swelling and pyroptosis. At the same time, caspase‐1 cleaves the precursors of IL‐1β and IL‐18 to be mature IL‐1β and IL‐18, which are released through the pores formed by N‐GSDMD.^28^, ^32^

#### 1.2.2 Non‐canonical pathway

The non‐canonical pathway is also named caspase‐1‐independent pathway. Cytosolic LPS, an activator of non‐canonical pyroptosis, directly binds and activates caspase‐4/5/11 protein to cleave GSDMD into N‐GSDMD,^10^, ^33^ inducing the formation of GSDMD pores and pyroptosis. Interestingly, although caspase‐4/5/11 cannot cleave pro‐IL‐1β and pro‐IL‐18 directly, they are capable of mediating the maturation and secretion of IL‐1β and IL‐18 through the NLRP3/caspase‐1 pathway.^10^, ^32^ In addition, caspase‐11 activated by cytosolic LPS cleaves pannexin‐1. Subsequently, ATP release and K+ outflow occur. They stimulate the P2X7 receptors and ion channels on their own membrane, inducing the assembly of NLRP3 inflammasomes and pyroptosis.^34^, ^35^, ^36^ However, murine BMDMs deficient in pannexin‐1 and P2X7 receptor could also cause K+ efflux and NLRP3 inflammasome‐mediated caspase‐1 cleavage.^35^, ^37^ The mechanism is still unknown.

#### 1.2.3 Other pathways

Apoptosis‐related caspases (such as caspase‐3/8) were thought to be unable to stimulate gasdermin to induce pyroptosis until it was shown that TNF‐α, chemotherapy drugs^9^ and granzyme B (GZMB)^38^ could induce caspase‐3‐mediated GSDME cleavage in high GSDME expression and form N‐GSDME termini and “gasdermin channels”, which caused pyroptosis in tumor cells. Knocking out GSDME switched lobaplatin‐induced cell death from pyroptosis to apoptosis.^39^ Additionally, caspase‐8 acts as a regulator of GSDMD‐driven cell death. Pathogenic Yersinia inhibited TAK1 via the effector YopJ and then activated RIPK1 and caspase‐8, forming the N‐GSDMD channels and N‐GSDME channels on the cell membrane and leading to the pyroptotic‐inflammatory response.^40^, ^41^ Besides, PD‐L1 could convert TNF‐mediated apoptosis into pyroptosis in breast cancer cells. Under the stimulation of both TNF‐α and PD‐L1, caspase‐8 specifically cleaved GSDMC to N‐GSDMC and formed the “gasdermin channels” on the cell membrane. Antibiotic chemotherapy drugs can also induce caspase‐8/GSDMC‐mediated pyroptotic death in breast cancer cells.^42^

**FIGURE 1:**
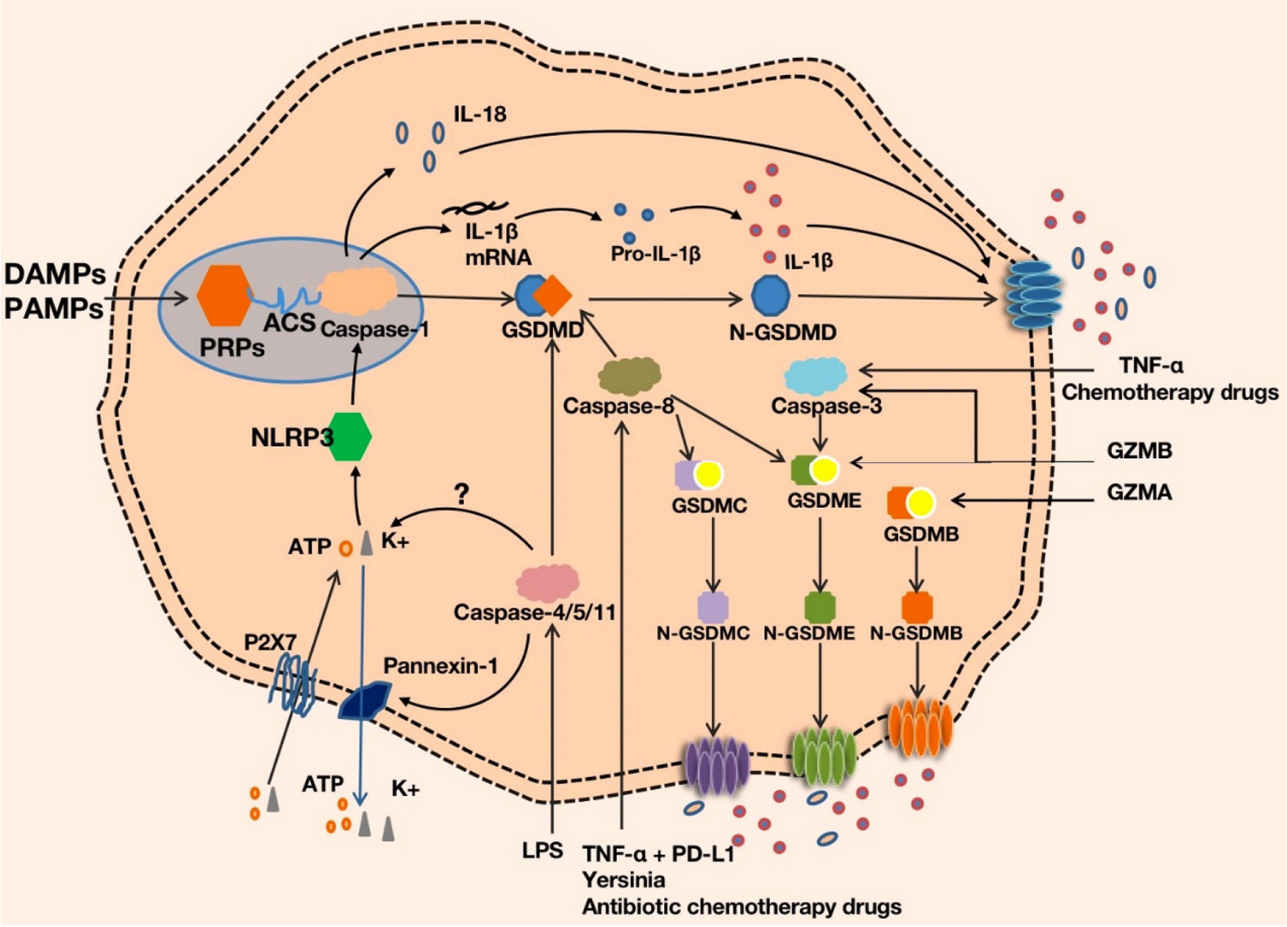
Mechanism of cell pyroptosis. Canonical pathway: after recognizing DAMPs or PAMPs, PRPs assemble with ASC and pro‐caspase‐1 to form inflammasomes and active caspase‐1, inducing IL‐1β and IL‐18 synthesis and the cleavage of GSDMD into N‐GSDMD. N‐GSDMD forms pores in membrane, leading IL‐1β and IL‐18 to release and cell swelling ultimately. ROS of the mitochondrion is one of the important factors to active inflammasomes. Non‐canonical pathway: ① LPS activates caspase‐4/5/11 protein to directly cleave GSDMD into N‐GSDMD or to active NLRP3 and caspase‐1 to induce the cleavage of GSDMD after K+ efflux and ATP release via pannexin‐1 and P2X7 or not; ② Caspase‐3 cleave GSDME induced by TNF‐α, chemotherapy drugs or GZMB; ③ Caspase‐8 cleave GSDMD and GSDME induced by Yersinia; ④ Caspase‐8 cleave GSDMC induced by TNF‐α/PD‐L1 and antibiotic chemotherapy drugs; ⑤ GZMB directly cleave GSDME; ⑥ GZMA directly cleave GSDMB.

In addition to caspase‐dependent pathways, the latest studies discovered that caspase‐independent GZMB directly cleaved GSDME and GZMA directly cleaved GSDMB, which unleashed its pore‐forming activity and redefined the idea that pyroptosis could only be activated by caspases.^43^ (Figure 1).

## 2 PYROPTOSIS IN PLATELETS

### 2.1 Inflammasome activation

Inflammasome components and assembly have not been shown in platelets until Hottz et al.^6^ reported preliminary RNA sequencing analyses, which indicated that megakaryocytes and platelets had components of the NLRP3 inflammasomes in 2013. In that study, flow cytometric analysis of intracellular NLRP3 confirmed that NLRP3 protein was present in platelets from both dengue‐infectious patients and healthy controls. Upon NLRP3 activation, NLRP3 inflammasomes were formed with ASC and pro‐caspase‐1, regulating the production and release of IL‐1β–rich microparticles. Further studies on the mechanism of platelet inflammasomes were reported. In the same year, Toll‐like receptors 4 (TLR4) activated by the LPS inplatelet membrane was found to be a critical protein upstream of NLRP3 inflammasomes.^44^ The caspase‐1 inhibitor Z‐WEHD‐FMK or the less selective inhibitor Z‐YVAD‐FMK blocked LPS stimulation of platelet pro‐IL‐1β hnRNA splicing. The role of TLR4 in platelets was further confirmed in sickle cell anemia patients, where HMGB1/TLR4 signaling increased platelet NLRP3 inflammasome activity in affected patients,^45^ as well as in sepsis patients, where GSDMD‐dependent platelet pyroptosis induced NET formation via S100A8/A9 and TLR4.^46^ Another study found that activation of platelets by collagen or thrombin promoted platelet NLRP3 inflammasome activation, which was found to be a critical regulator of platelet activation, aggregation, and in vitro thrombus formation.^47^

Reactive oxygen species (ROS) was reported to be a critical mechanism triggering the assembly of NLRP3 inflammasomes in response to DAMPs from damaged cells in human macrophage cells, and mitochondria were the main source in mammalian cells.^48^ For platelets, ROS was only connected to platelet activation and thrombus formation before the discovery of inflammasomes in platelets.^49^, ^50^, ^51^ In 2013, mitochondrial‐derived ROS generation was first reported to increase along with NLRP3 inflammasome activation in platelets.^6^ H_2_O_2_‐treated platelets, which induced ROS generation, elevated the expression of NLRP3 inflammasomes and increased IL‐1β secretion. And, these high levels of intracellular ROS were observed in NLRP3 inflammasome‐activated platelets in Crohn's disease,^52^ immune thrombocytopenia(ITP)^53^ and sepsis.^46^ The presence of the mitochondrial targeted antioxidant mitoTEMPO reduced mitochondria‐generated ROS, caspase‐1 activation, and IL‐1β secretion in these diseases.^6^, ^54^ In addition, receptor‐interacting protein 1 and 3 (RIP1/RIP3) kinases have been shown to be regulators of NLRP3‐dependent caspase‐1 and IL‐1β activation by promoting mitochondrial ROS production. Exposure of platelets to dengue virus in the presence of RIP‐1/RIP‐3 inhibitor Nec‐1 prevented the generation of mitochondrial‐derived ROS.^6^ However, loss of RIP1/RIP3 had no impact on alum‐, R837‐, ATP‐, or nigericin‐induced NLRP3 activity, demonstrating that RIP1/RIP3 was not required for optimal NLRP3 activation by other stimuli.^55^

Bruton's tyrosine kinase (BTK) has been identified as an essential regulator of the NLRP3 inflammasomes in innate immune cells in recent years.^56^, ^57^ In fact, L.S. Quek et al.^58^ found that BTK was expressed by platelets and was important for signaling via collagen receptor glycoprotein VI (GPVI) as early as 1998. In 2017, Murthy et al.^47^ showed that platelet NLRP3 inflammasome activation, as monitored by caspase‐1 activation and cleavage and secretion of IL‐1β, was dependent on platelet BTK, which provided one possible explanation for the clinical observation that patients treated with the BTK inhibitor ibrutinib may experience increased bleeding events and reduced platelet aggregation.Coincidentally, the authors in the study proposed whether the platelet NLRP3 inflammasomes were involved in regulating pyroptosis‐like changes in platelets, while no further research was conducted. In addition, Vogel et al.^45^ showed that regulated by HMGB1/TLR4 and BTK, the NLRP3 inflammasomes were upregulated in platelets from sickle cell anemia patients and sickle cell mice. The BTK inhibitor ibrutinib decreased platelet NLRP3 inflammasome activation and platelet aggregation in this study, confirming BTK as a novel regulator of NLRP3 activation in platelets.

The autocrine and paracrine signaling loop of IL‐1β is a critical way to activate platelets. Platelets were anucleate blood cells that were not thought to synthesize proteins or cytokines or to influence inflammatory responses in the past. As early as 1989, Hawrylowicz et al.^59^ have reported that stimulated platelets possessed IL‐1β, but they were not thought to actively synthesize this protein. In 1993, Kaplanski et al.^60^ showed that activated platelets could induce IL‐8 secretion of endothelial cells via membrane‐associated IL‐1 activity. In 2001, Lindemann et al.^61^ confirmed that quiescent platelets contained many messenger RNAs by searching from an arrayed cDNA library. One of mRNAs coded for interleukin‐1β precursor (pro–IL‐1β), IL‐1β and many other transcripts were constitutively present in polysomes, providing a mechanism for rapid synthesis.^62^ They identified that platelets activated with thrombin (IIa) induced rapid and sustained synthesis of pro‐IL‐1β protein during fibrin clot formation, and the progress was abolished by translational inhibitors (puromycin).^63^ Interestingly, in addition to secreting IL‐1β, platelets expressed IL‐1 receptor 1 by themselves. It implied platelets could respond to the IL‐1β they made and IL‐1β could stimulate splicing and translation of the newly made IL‐1β mRNA in platelets. Specific blockade of IL‐1R1 with IL‐1 receptor antagonist (IL‐1Ra) suppressed platelet stimulation by IL‐1, so IL‐1β stimulated its own synthesis in an autocrine and paracrine signaling loop. Besides, IL‐1β synthesis and its subsequent stimulation of IL‐1R1 were essential components for platelet activation by LPS.^44^

Inflammasomes, one of the most important parts of pyroptosis, were gradually confirmed in platelets. Not surprisingly, IL‐18, another critical cytokine in pyroptosis, was found to be transcribed in human platelets and platelet activation increased IL‐18 concentrations in the circulation for the first time in 2017.^64^

However, there are still controversies in this field. Rolfes et al.^2^ reported that stained and assessed by flow cytometry and confocal microscopy, ASC was visualized and assembled into fluorescent specks in inflammasome‐activated leukocytes, macrophages, and neutrophils, but not in platelets and MKs of mouse bone marrow (BM) cells and in human isolated platelets after activation by LPS. Furthermore, they did not detect the expression of NLRP3, ASC, and caspase‐1 and IL‐1β at the mRNA and protein level, which might be due to different experimental conditions and readouts.

### 2.2 Pyroptotic detection in platelets

Annexin V bonds with high affinity to negatively charged phospholipids like phosphatidylserine (PS) of the extracellular membrane when cells die,^65^ as pores open in the cell membrane, permitting annexin V to enter and stain the inner membrane leaflet. As there was no specific molecule for pyroptosis in the past, Mao et al. thought caspase‐1 and annexin V double‐positive could be used to detect pyroptosis.^66^ Wang et al.^53^ considered caspase‐1+/annexin V− cells to be early pyroptotic cells where pores have not formed in the cell membrane, preventing annexin V from entering cell and staining, and caspase‐1+/annexin V+ as late pyroptotic cells. And, they reported that the proportion of caspase‐1+/annexinV+ and caspase‐1+/annexinV− pyroptotic platelets was increased with H_2_O_2_ treatment, which induced NLRP3 inflammasome activation. However, caspase‐1+/annexin V+ was detected in both apoptotic and pyroptotic cells, which means that it is not specific to pyroptosis.^67^ Whether platelets could undergo pyroptosis was still controversial.

**FIGURE 2:**
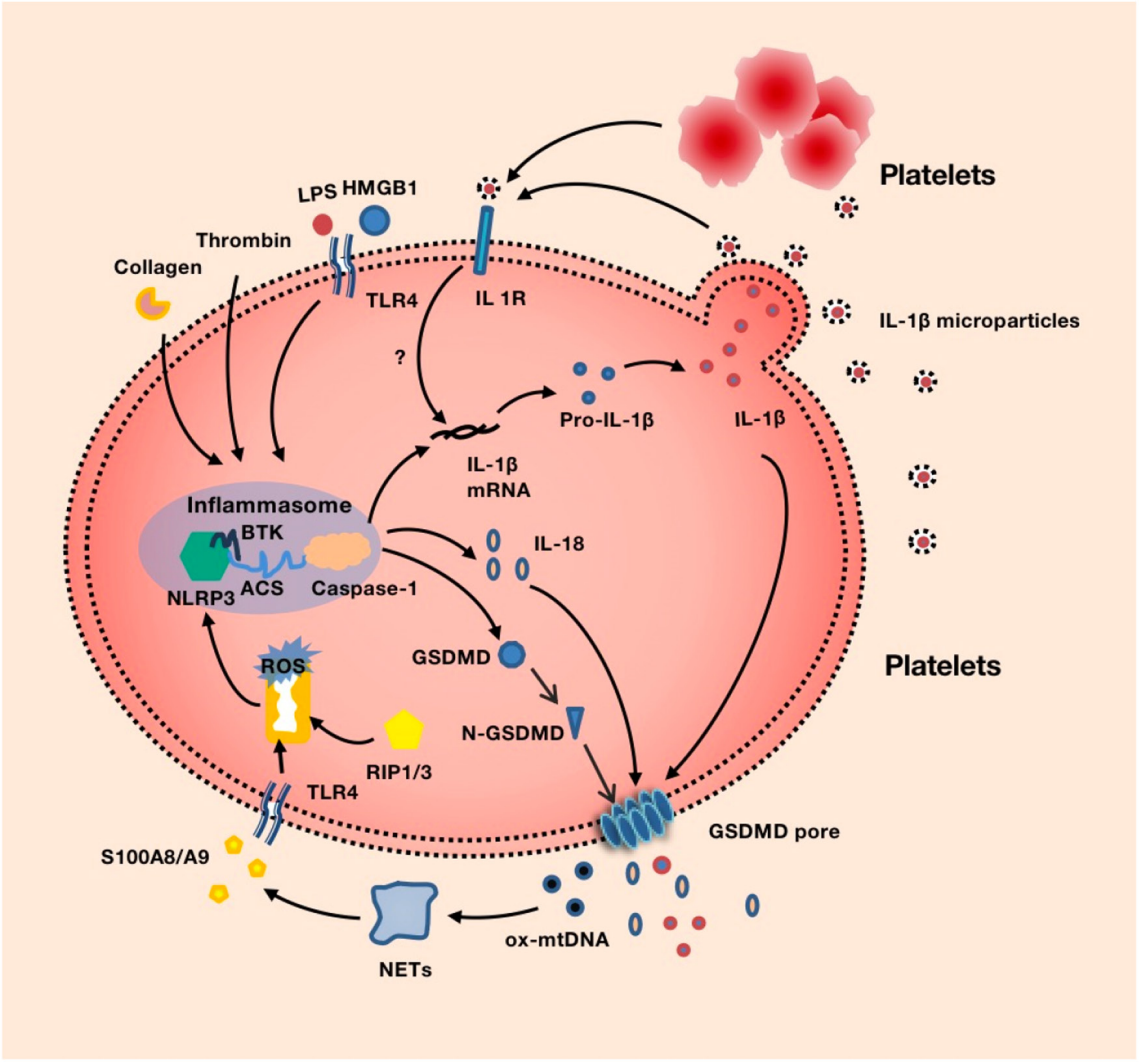
Mechanism of platelet pyroptosis. Platelet activation via LPS, S100A8/A9 and HMGB1 receptor TLR4, collagen receptor GPVI, and thrombin stimulate NLRP3. NLRP3 formed inflammasomes with the ASC, BTK, and pro‐caspase‐1 that regulated the production of proinflammatory cytokines such as IL‐1β and IL‐18. IL‐1β stimulates its own synthesis in an autocrine and paracrine signaling loop via IL‐1R1. Upon NLRP3 inflammasome activation, GSDMD is activated by caspase‐1. Mitochondrial‐derived ROS and its upstream of RIP1/RIP3 regulate NLRP3 inflammasome recruitment. Pyroptotic platelet‐derived oxidized mitochondrial DNA (ox‐mtDNA) potentially promotes neutrophil extracellular trap (NET) formation, which contribute to platelet pyroptosis by releasing S100A8/A9, forming a positive feedback loop that led to the excessive release of inflammatory cytokines.

After Shao's study in 2015, GSDMD was considered to be a specific marker to detect pyroptosis. In 2016, Lien et al.^68^ showed that pyroptosis was the main cell death way in DENV and Envelope Protein Domain III (rEIII)‐induced platelets and was suppressed by the pyroptosis/GSDMD inhibitor disulfiram (DMF). Additionally, cleaved GSDMD was detected via Western blot. Unfortunately, there is no more precise morphological report to certify the pyroptosis in platelets. By 2022, Su et al. reported GSDMD and cleaved GSDMD were significantly increased in platelets of sepsis patients and CLP mice. Importantly, they first observed rapid swelling and membrane rupture in mouse platelets after LPS and nigericin (NIG) treatment, but these effects were absent in the GSDMD‐KO platelets, providing strong evidence for pyroptosis in platelets. Besides, they found GSDMD‐dependent platelet pyroptosis was induced by high levels of S100A8/A9 targeting TLR4. Pyroptotic platelet‐derived oxidized mitochondrial DNA (ox‐mtDNA) potentially promoted neutrophil extracellular trap (NET) formation, which contributed to platelet pyroptosis by releasing S100A8/A9, forming a positive feedback loop that led to the excessive release of inflammatory cytokines^46^ (Figure 2).

## 3 PYROPTOSIS IN PLATELETS AND DISEASES

Activated platelet NLRP3 inflammasomes have been confirmed in many diseases such as ulcerative colitis,^69^ Crohn's disease,^52^ hindlimb ischemia,^70^ acute coronary syndrome,^71^ pancreatic cancer^72^ and sickle cell disease.^73^ Usually, inflammasome‐dependent shedding of IL‐1β and caspase‐1‐carrying platelet EVs will contribute to platelet aggregation, vaso‐occlusion, endothelium permeability, and inflammation, regulating thromboinflammation. Once GSDMD‐pore is formatted and all cellular contents efflux out of the cell nonselectively, more serious clinical outcomes will occur, including excessive inflammation and thrombocytopenia.However, not all diseases in which platelet inflammasomes are activated have been confirmed to induce pyroptosis. Here, we conclude the three confirmed diseases.

### 3.1 Dengue

Dengue is a mosquito‐borne infectious disease triggered by dengue virus (DENV). Dengue is the most frequent hemorrhagic viral disease and reemergent infection in the world. Over half of the global population lives in areas with DENV infection risk, resulting in 96 million patients with symptomatic dengue every year.^74^ DENV infection leads to several clinical symptoms, including dengue fever (DF), which is a kind of asymptomatic mild flu that can enhance vascular fragility, and thrombocytopenia, well‐known as Dengue Hemorrhagic fever (DHF), which leads to hypovolemic shock called Dengue Shock Syndrome (DSS), a more severe condition.^75^, ^76^ Although dengue pathogenesis remains elusive, the cytokine storm has been considered to be one of the primary and crucial causative factors.^77^ Inflammasome assembly and IL‐1β secretion in innate immune cells are the main factors.^78^

DENV infection leads to cell death of platelets and megakaryocytes. Thus, thrombocytopenia and platelet defects are commonly observed in mild and severe dengue syndromes. With the exploration of platelet immune function, it is easy to connect the cytokine storm with platelets. In 2013, Hottz et al.^6^ found for the first time that DENV infection led to assembly of NLRP3 inflammasomes, activation of caspase‐1, and caspase‐1–dependent IL‐1β secretion in platelets. Platelet‐derived IL‐1β was mainly released in microparticles through mitochondrial ROS–triggered NLRP3 inflammasomes, contributing to increased vascular permeability. In 2021, Lien et al.^68^ found that DENV and virion‐surface envelope protein domain III (EIII), a cellular binding moiety of the virus particle, induced NLRP3 inflammasome activation and GSDMD cleavage. Therefore, pyroptosis is the major regulated cell death (RCD) pathway of DENV‐ and EIII‐treated platelets. NLPR3 inflammasome components and GSDMD may be feasible targets for treating dengue‐associated thrombocytopenia and platelet defects.

### 3.2 Immune thrombocytopenia (ITP)

Immune thrombocytopenia (ITP) is an acquired thrombocytopenia characterized by a low platelet count and increased risk of bleeding. Their bleeding tendency ranges from petechiae and purpura to intracranial hemorrhages.^79^ Accelerated T‐cell‐mediated immune destruction of platelets and impaired production of platelets are the two main causes of thrombocytopenia in ITP.^80^ NLRP3 inflammasomes play an important role in the regulation of the adaptive immune response, especially in T‐cell response. Given the close association of an imbalance in T‐cell response with ITP, the expression of NLRP3 and ASC in patients with active ITP has been confirmed to be significantly higher than that in patients with resting ITP.^81^, ^82^

Growing evidence has proven the importance of platelets as both immune sensing and immune effector cells in innate and adaptive immunity. ITP is closely related to inflammation as an infection. Both viral and bacterial factors can trigger ITP, and many proinflammatory mediators are involved in ITP. Binding of bacterial LPS to platelet TLR4 has been shown to induce thrombocytopenia in mice in the presence of anti‐platelet antibodies.^83^ Therefore, we presume that NLRP3 inflammasomes are not only present in T cells but also expressed in platelets in ITP patients. Wang et al.^53^ found that ITP platelets showed a higher expression and activation of the NLRP3 inflammasomes and were more vulnerable to oxidative stress because of ITP‐related reduced intracellular antioxidant capacity. Subsequently, platelet pyroptosis was induced, which was an important pathway to low platelet levels in the circulation.

### 3.3 Sepsis

Sepsis is a complex syndrome characterized by organ dysfunction and a dysregulated immune host response to infection.^84^ There is currently no effective treatment for sepsis, but platelets have been proposed as a potential therapeutic target. The contribution of platelets to sepsis progression goes beyond thrombosis and coagulation. Platelets have emerged as major drivers of the innate and adaptive immune responses. Septic patients with increased platelet activation and low platelet count are prone to develop multiple organ dysfunction and have increased 90‐day mortality.^85^

Associations with platelet activation and sepsis severity are clearly demonstrated clinically, and both preclinical and clinical studies show improved outcomes with antiplatelet therapy in sepsis. However, further molecular mechanism was unclear. In 2019, Denise et al. reported for the first time that NLRP3 was confirmed to be activated in platelets stimulated by LPS or sepsis in CLP rats.^86^ In 2020, they further discovered that treatment with MCC950, a specific NLRP3 inhibitor, attenuated NLRP3 activation in platelets as well as multi‐organ injury in CLP rats.^87^ And, in 2022, platelet pyroptosis was associated with sepsis. Platelet proteomic analysis revealed significant upregulation of GSDMD. Using platelet‐specific GSDMD‐deficient mice, Su et al. demonstrated a requirement for GSDMD in triggering platelet pyroptosis in CLP‐induced sepsis. GSDMD‐dependent platelet pyroptosis was induced by high levels of S100A8/A9 targeting TLR4. Pyroptotic platelet‐derived oxidized mitochondrial DNA (ox‐mtDNA) potentially promoted neutrophil extracellular trap (NET) formation, which contributed to platelet pyroptosis by releasing S100A8/A9, forming a positive feedback loop that led to the excessive release of inflammatory cytokines. Both pharmacological inhibitions using paquinimod and genetic ablation of the S100A8/A9–TLR4 signaling axis improved survival in mice with CLP‐induced sepsis by suppressing platelet pyroptosis.^46^

## 4 FUTURE DIRECTIONS

The role of platelets in infectious and sterile inflammation is increasingly being confirmed. Thrombocytopenia is observed in some infectious diseases such as dengue,^6^ novel coronavirus disease (COVID‐19)^88^ and sepsis^46^ and sterile diseases such as ITP,^53^ disseminated intravascular coagulation (DIC)^89^ and post‐ischemic reperfusion (myocardial infarct, stroke, and acute renal injury). Severe thrombocytopenia progression goes beyond thrombosis and coagulation. Platelets have emerged as major drivers of the innate and adaptive immune responses. With the activation of caspase‐1 and the release of inflammatory cytokines such as IL‐1β and IL‐18, one of the hypotheses of thrombocytopenia is that pyroptosis is one of the cell death pathways of platelets, which may be an important intervention and therapeutic target of inflammation. Inhibitors of these interactions might serve as powerful anti‐inflammatory agents without impairing hemostasis.While NLRP3 inflammasomes in platelets have been recently described, the role of many other inflammasomes remains to be elucidated. Although pyroptosis in platelets has been reported in several studies,^6^, ^46^, ^53^ more mechanisms and regulatory factors are needed to research.

## 5 CONCLUSION

The classical view of platelets restricted to hemostasis has been left. Indeed, numerous studies have demonstrated how platelets tightly regulate inflammation through recruitment and activation of immune cells, release of proinflammatory factors and direct interactions with invading pathogens. However, pyroptosis was only recently described in platelets, which opened new perspectives and research opportunities in disease pathogenesis. As discussed above, platelet NLRP3 activation is induced via LPS, HMGB1 and S100A8/A9 receptor TLR4, collagen receptor GPVI, and thrombin. Upon NLRP3 activation, NLRP3 forms inflammasome complexes with ASC and pro‐caspase‐1 that regulate the production of proinflammatory cytokines such as IL‐1β and IL‐18, at the same time, cleaving GSDMD and inducing pyroptosis. IL‐1β stimulates its own synthesis in an autocrine and paracrine signaling loop via IL‐1R1. Mitochondrial‐derived ROS and its upstream of RIP1/RIP3 regulate NLRP3 inflammasome recruitment. Pyroptotic platelet‐derived oxidized mitochondrial DNA (ox‐mtDNA) potentially promotes neutrophil extracellular trap (NET) formation, which contributes to platelet pyroptosis by releasing S100A8/A9, forming a positive feedback loop that leads to excessive release of inflammatory cytokines.Although NLRP3 inflammasomes and pyroptosis have been demonstrated in platelets, there is little information regarding pyroptosis in platelets. Conditions in which platelet inflammasomes and pyroptosis play pathogenic and/or protective roles in inflammation and immune response still deserve more in‐depth investigation.

## AUTHOR CONTRIBUTIONS

All the authors have accepted responsibility for the entire content of this submitted manuscript and approved submission. Yang Su wrote the first draft and contributed to all revisions of the manuscript; other authors highlighted important articles, reviewed the first draft, and contributed to the final version.

## FUNDING INFORMATION

This study was supported by the Natural Science Foundation of China (Grant No. 82072352) and Natural Science Foundation of Beijing Municipality (Grant No. 7192222).

## CONFLICT OF INTEREST STATEMENT

The authors declare that they have no competing interests.

## Data Availability

Data sharing not applicable to this article as no datasets were generated or analysed during the current study
